# Effect of the Alloying Metal on the Corrosion Resistance of Pd-Rich Binary Alloys with Pt, Rh, and Ru in Sulfuric Acid

**DOI:** 10.3390/ma14112923

**Published:** 2021-05-28

**Authors:** Katarzyna Hubkowska, Julian Kubisztal, Małgorzata Pająk, Bożena Łosiewicz, Andrzej Czerwiński

**Affiliations:** 1 Faculty of Chemistry, University of Warsaw, Pasteura 1, 02-093 Warsaw, Poland; mpajak@chem.uw.edu.pl (M.P.); aczerw@chem.uw.edu.pl (A.C.); 2 Institute of Materials Engineering, Faculty of Science and Technology, University of Silesia in Katowice, 75 Pulku Piechoty 1A, 41-500 Chorzow, Poland; julian.kubisztal@us.edu.pl (J.K.); bozena.losiewicz@us.edu.pl (B.Ł.)

**Keywords:** Pd, Pd-Rh alloy, Pd-Ru alloy, Pd-Pt alloy, electrodeposition, corrosion resistance, SKP, work function, fermi level

## Abstract

The paper presents the study of the corrosion resistance of electrodeposited Pd and its binary alloys with Pt, Rh, and Ru on a polycrystalline Au substrate. The corrosion resistance was tested in 0.5 M sulfuric acid at room temperature using potentiodynamic polarization and electrochemical impedance spectroscopy techniques. The morphology/composition and work function values were determined by scanning electron microscopy/energy–dispersive X–ray spectroscopy and scanning Kelvin probe, respectively. The obtained results revealed that the Pd electrode is the most resistant to corrosion, whereas the Pd-Ru electrode is the most susceptible to dissolution. It was found that the corrosion resistance of Pd-binary alloys decrease in the following order: Pd > Pd-Pt > Pd-Rh > Pd-Ru. This effect was assigned mainly to the difference in surface roughness factor of tested electrodes.

## 1. Introduction

In recent times, palladium and its alloys with other noble metals have been intensively studied as they can improve the chemical and mechanical properties of metal coatings. It is especially important for industrial applications, where protective coatings are applied to enhance the corrosion resistance of bare metal. Pd/Ag alloys are used in dentistry as they are biocompatible and have high distortion resistance during porcelain firing [1]. They are also used as membranes for hydrogen separation and hydrogen storage materials [2,3]. Palladium alloyed with Ni, Co, or Cu is used to obtain cathodic coatings on stainless steel as the noble metal enhances passivation and improves the corrosion resistance [4,5,6]. Pd/Ni alloys are mostly used for electrically conductive surfaces as they have an excellent wear resistance [7]. Au/Pd surface finish is also utilized in electronic industry to enhance solderability and oxidation resistance [8]. Pd-based binary and ternary alloys have recently been screened in electrocatalytic reduction of CO_2_ and O_2_ or formaldehyde and CO oxidation since they are comparatively inexpensive, have higher catalytic activity and lower sensitivity to poisoning effect in comparison with a platinum catalyst [9,10,11,12,13,14].

In the last decades, palladium and its alloys with Pt, Ru, and Rh have also been extensively studied in an aspect of hydrogen absorption phenomenon from both aqueous and non-aqueous electrolytes, as well as from the gas phase [15,16,17,18,19,20,21,22,23,24,25,26]. The addition of a second noble metal to palladium changes the properties of the alloy for hydrogen absorption. It is caused by alteration of the structure of crystal lattice, elastic and electronic properties. Consequently, it results in changes in the kinetics of the sorption process, the amount of the absorbed hydrogen, and the range of absorption/desorption hysteresis [21,27,28,29]. Pd and Pt form a solid solution, and its lattice parameters somewhat increase with Pt addition (expanded alloys) [30]. In the case of Pd-Ru and Pd-Rh alloys after the addition of alloying metal the decrease of lattice constant is observed and therefore, they are classified as contracted alloys [21,31]. Pd-rich Pd-Pt alloys absorb hydrogen electrochemically, similarly to pure Pd. At high Pd bulk content, hydrogen is absorbed in the β phase, while at a lower Pd content, the hydrogen is absorbed in the α phase. The ability of the Pd-Pt alloys to absorb hydrogen decreases with increasing Pt bulk content [32]. It is caused by the effect of the valence band broadening upon alloying [30]. On the contrary, in Pd-Ru alloys, in a certain composition range, the increased absorption capacities are observed [20,29]. It is due to the fact that Ru possesses free states around the Pd Fermi level. The amount of absorbed hydrogen increases with the increasing Ru bulk content, and alloys containing 98–99.5 at.% of Pd absorb more hydrogen than pure Pd [20]. Among various Pd alloys, the best absorption properties show Pd-Rh systems, and the highest amount of the absorbed hydrogen is observed for alloys containing ≤20 at.% of Rh. Doping the palladium with rhodium increases the amount of 4d band vacancies, facilitating the transfer of hydrogen to this band, and the solubility of hydrogen in the β hydride phase is larger than in Pd [16].

The well-known and uncomplicated method for the production of numerous alloys is electrodeposition. The morphology, composition, microstructure, physicochemical properties, and thickness of the electrodeposits can be controlled by the deposition techniques and their parameters, i.e., current, voltage, temperature, or bath composition [33]. Since in Pd-Ru and Pd-Rh systems large miscibility gap is observed, it should be taken into account when selecting the method of preparation of the alloys [34,35].

Despite extensive studies on Pd-noble metals alloys’ ability to hydrogen absorption, there is a lack of information about the corrosion resistance of such alloys. Hence, this study aims to investigate the corrosion behavior of three Pd-noble metal electrodeposited alloys, i.e., Pd-Ru, Pd-Rh, and Pd-Pt. Since previously conducted research on the hydrogen electrosorption in Pd-based alloys were carried out in 0.5 M sulfuric acid, this medium has been chosen in corrosion experiments. The DC method of potentiodynamic polarization and the AC method of electrochemical impedance spectroscopy (EIS) were used. The obtained results were compared with those for the pure Pd electrodeposit.

## 2. Materials and Methods

### 2.1. Material Preparation

The Pd-noble metal alloys were prepared by potentiostatic electrodeposition on a polycrystalline gold plate (0.12 mm thick, 99.99%, Mint–Metals, Radzymin, Poland) from baths containing water solutions of 0.11 M PdCl_2_ acidified with 1 M HCl and 0.05 M H_2_PtCl_6_, 0.32 M RuCl_3_, or 0.5 M RhCl_3_, separately, depending on the desired alloy. The thickness of electrodeposits was ca. 1 µm. It was confirmed by the calculations based on the deposition charge and deposition efficiency designated previously [19,21,31]. All solutions were prepared from analytical grade reagents and ultra–pure water (Millipore, Burlington, MA, USA). Detailed physicochemical characteristics of electrodeposited Pd-based electrodes can be found in [36]. The formation of the alloys was confirmed previously [31,36] by XRD analysis and simultaneously for hydrogen-absorbing alloys by the change of the α→β phase transition potential compared to pure Pd [19,21,31].

### 2.2. Electrochemical Measurements

The electrochemical measurements were conducted in 0.5 M H_2_SO_4_ deaerated with argon (99.999%, Air Products, Warsaw, Poland) for 30 min. During experiments, an argon stream was directed over the solution. A three–electrode system was used with a thin layer alloy as a working electrode, Hg|Hg_2_SO_4_|0.5 M H_2_SO_4_ as a reference electrode, and Pt gauze as an auxiliary electrode. All electrochemical measurements were performed by means of CH Instruments Electrochemical Workstation (CHI 760d, Inc., Austin, TX, USA). The potentials were recalculated in reference to the reversible hydrogen electrode (RHE).

EIS measurements were performed at open circuit potential (*OCP*) using AC amplitude of 10 mV, and frequencies range from 10 kHz to 5 mHz (10 frequencies per decade). EIS spectra were analyzed by the complex non-linear least squares (CNLS) method using ZSimpWin 3.21 software (EChem, Ann Arbor, MI, USA). Potentiodynamic polarization curves were registered at a potential range of ±200 mV against *OCP*. The analysis of the experimental EIS data was carried out using the selected equivalent electrical circuits.

### 2.3. Material Characteristics Methods

The bulk composition of the Pd-based alloys was determined by energy–dispersive X-ray spectroscopy (EDX, Quantax 400 energy dispersive X-ray spectroscope, Bruker, Billerica, MA, USA). The surface morphology observations were performed using scanning electron microscopy (SEM, Merlin field emission scanning electron microscope, Zeiss, Germany). The contact potential difference (*CPD*) maps of electrodeposits were recorded by scanning Kelvin probe (SKP) using PAR Model 370 Scanning Electrochemical Workstation (Princeton Applied Research, Oak Ridge, TN, USA) equipped with a tungsten SKP microprobe (Ø 500 μm, Princeton Applied Research, Oak Ridge, TN, USA). 

## 3. Results and Discussion

### 3.1. Morphology and Bulk Composition of the Pd-Alloys before and after Corrosion

**Figure 1:**
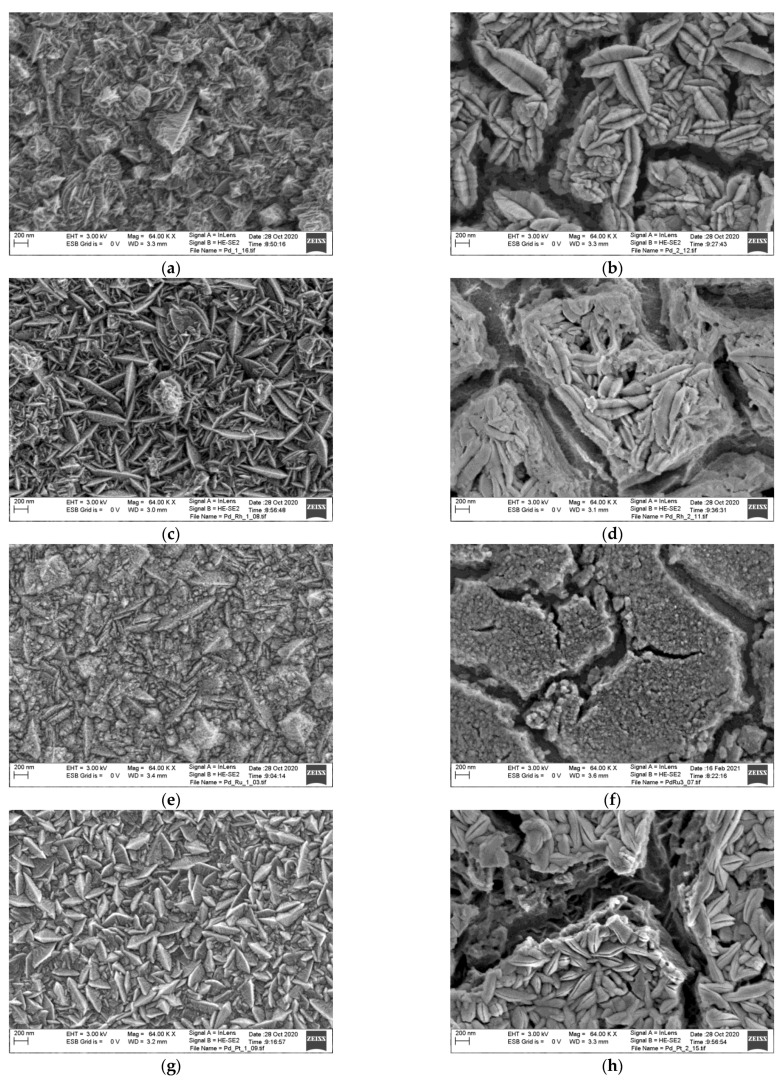
Surface morphology of the electrodeposited Pd and Pd-based alloys: (**a**) As–deposited Pd; (**c**) As–deposited Pd-Rh; (**e**) As–deposited Pd-Ru; (**g**) As–deposited Pd-Pt; (**b**) Pd after corrosion; (**d**) Pd-Rh after corrosion; (**f**) Pd-Ru after corrosion; (**h**) Pd-Pt after corrosion.

The electrodeposition of the Pd-rich (below 5 at.% of alloying metal: see Table 1) Pd-binary alloys with Rh, Ru, and Pt leads to obtaining grey and smooth layers. As–deposited Pd and Pd-based alloys are uniform and free of cracks (Figure 1a,c,e,g). The electrodeposits’ surface morphology generally resembles the one for the pure Pd, where star–like crystallites are present. The surface morphology of Pd-Rh alloy is similar to pure Pd; however, it is more lamellar. In the case of Pd-Ru alloys, lamellas and finer round crystallites are visible at the surface. The surface morphology of the Pd-Pt alloy is also lamellar but with crystallites similar in size, bigger than in the case of Pd and other studied alloys.

After applying the corrosion experiment using the potentiodynamic technique, the morphology of the Pd and its alloys have been changed. Many cracks are visible at the surface of all studied samples (Figure 1b,d,f,h). Moreover, the corrosion process results in a degradation of lamellas in the areas between the cracks. Small particles form more amorphous structures, whereas larger lamellar-shaped particles tend to break into parts or merge into larger lips-like structures. However, the Pd-Ru alloy seems to be an exception to above discussion since after the corrosion (Figure 1f), only amorphous morphology is visible. It is mostly due to the fact that the particles at the surface of as-deposited Pd-Ru alloys are the smallest among all examined alloys.

For the Pd-Rh alloys in the areas between the cracks, more amorphous structures are present, indicating a higher rate of corrosion. The morphology of Pd-Ru alloys after corrosion differs from the morphology of other electrodes. In the case of Pd-Ru, the cracks of various sizes are visible: larger cracks than for other electrodes and more minor cracks inside the areas limited by larger ones. Moreover, the crystallites’ size significantly diminishes, indicating that all surface has been involved in the dissolution process.

EDX measurements revealed that Pd binary alloys with similar content of the different alloying metal (Table 1) can be obtained by the tuning the electrodeposition potential and ions concentrations in galvanic baths. After the corrosion experiment, the bulk composition of the alloys was determined again. One can note that no changes in the bulk composition occurred for the Pd-Rh and Pd-Ru alloys. However, for the Pd-Pt alloys, slight enrichment (ca. 2 at.%) in Pt is observed.

Pd-Pt, Pd-Rh [37], and Pd-Ru [38] alloys have been previously tested in relation to their resistance to electrochemical dissolution. The alloy electrodes with different compositions were cycled to various upper limits of potentials (0.3 ÷ 1.5 V vs. RHE) with a scanning rate of 100 mV s^–1^. Cyclic voltammetry measurements were coupled with electrochemical quartz crystal microbalance (EQCM). In the case of every tested type of alloy, it was found that alloying Pd with Rh, Ru, and Pt results in the increase of the resistance to electrochemical dissolution in described experimental conditions. It was reported by the decrease of the ∆*m*/*S*, which described the change of the electrode mass (determined from EQCM) divided by the real surface area. The use of EQCM also enabled the calculation of the oxidation state (*z*) of dissolving metals. For Pd and Pd-Pt alloys *z* value of 2 was obtained, indicating Pd dissolution to Pd^2+^ in both cases. This is in line with the composition of Pd-Pt alloy obtained after corrosion, where enrichment with Pt is observed. In the case of Pd-Rh, the value of *z* was calculated in the range of 2.7–3.3. Here, it was concluded that the *z* value around 3 for Pd-Rh alloy results from both Pd (*z* = 2) and Rh (*z* ≥ 3) dissolution. Long cycling of Pd-Rh electrode to the 1.5 V resulted in the electrode enrichment in Pd [37]. For Pd-Ru alloy, the calculated value of *z* equals ca. 2.6, confirming Ru ions’ dominant contribution in the process of Pd-Ru alloy dissolution [38]. It is worth noting that below 1 V, mainly Rh [39] and Ru [38], are the most soluble, while Pd dissolution occurs above ca. 1 V. To the above discussion and chemical composition after corrosion, it can be concluded that in the conditions of the potentiodynamic experiment, the amount of the dissolved Pd, Rh, and Ru in Pd-Rh and Pd-Ru alloys are comparable, in general resulting in the same composition than before the corrosion process.

### 3.2. Potentiodynamic Measurements

**Figure 2:**
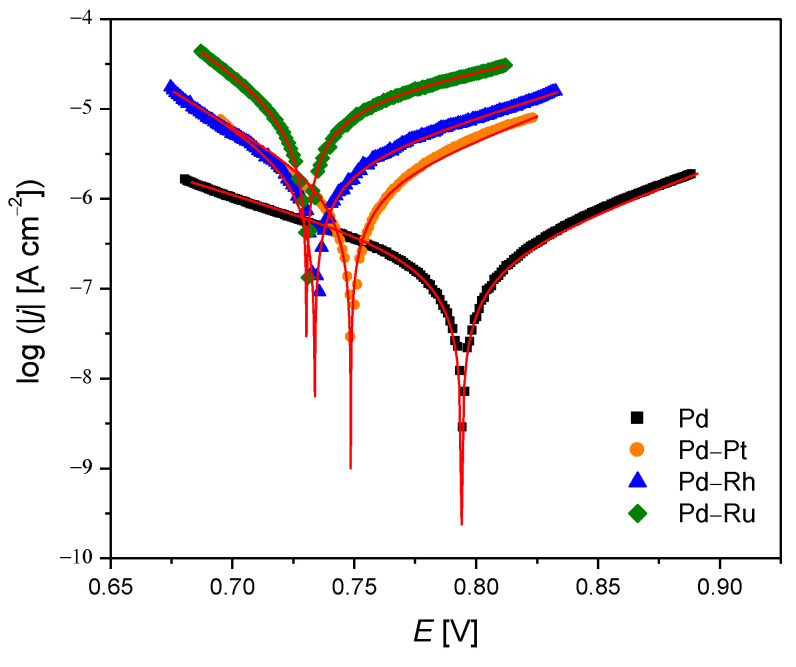
Potentiodynamic polarization curves obtained for electrodeposited Pd, Pd-Rh, Pd-Ru, and Pd-Pt electrodes in deaerated 0.5 M H_2_SO_4_. Symbols and continuous lines represent experimental data and fits obtained using the Butler–Volmer equation, respectively.

The results calculated from the potentiodynamic polarization curves shown in Figure 2 are placed in Table 2. The obtained values of the corrosion current (*j_cor_*) and corrosion potential (*E_cor_*) indicate that pure Pd is the most resistant to corrosion. The Pd-Ru and the Pd-Rh alloys occurred to be the most susceptible to corrosion in an acidic medium. Pd-Pt alloy exhibits intermediate behavior between Pd and other alloys. For all the tested samples of Pd-binary alloys, the *b*_a_ Tafel slopes’ values are higher than *b*_c_ Tafel slopes indicate that the anode processes at the *E_cor_* are faster than the cathode processes. 

The pure Pd electrode exhibits the opposite behavior. The average value of polarization resistance (*R*_p_) is the highest for the Pd electrode and the lowest for the Pd-Ru alloy electrode. *R*_p_ value for the Pd electrode is ca. 47 times higher than for Pd-Ru alloy electrode. These values also confirm the highest corrosion resistance of Pd electrode and the lowest corrosion resistance of the Pd-Ru alloy. Since the values of corrosion rate (*CR*) are inversely proportional to *R*_p_ values, the highest value of *CR* was calculated for Pd-Ru alloy. The lowest value of *CR* is observed for the Pd electrode. Based on the obtained results, it can be concluded that Pd–based electrodes are resistant to corrosion as follows: Pd > Pd-Pt > Pd-Rh > Pd-Ru.

The corrosion resistance properties of the electrode are dependent on the surface state of the alloys. They are influenced not only by the type of the alloying metals but also by, e.g., oxidation states of the elements and surface roughness factor (*ƒ*). Table 3 shows the calculated ƒ values of the electrodeposited electrodes. The *ƒ* values were calculated based on charge originating from the surface oxides reduction, according to the following formula [40]:

(1) $$f=\frac{Q_{red}^{O}}{\theta_{O}Q_{red,ML,S\;}^{O}S}$$

where *θ_O_* is the surface coverage with surface oxide, *Q^O^ _red_*_,*ML*,*S*_ is the charge from the reduction of a monolayer of surface oxide per unit area, and *S* is the geometric surface area. Since the experiments have been done on Pd-rich alloys, the charge due to the reduction of a monolayer of surface oxide per unit area equals the same value as in the case of pure Pd, i.e., ca. 424 µC cm^–2^. In the condition of the experiment (polarization to 1.5 V vs. SHE, scan rate 0.1 Vs^−1^) it can be assumed that the oxide monolayer is formed (*θ_O_* equals to 1).

One can notice that Pd-Ru alloys exhibit the roughest surface, whereas the Pd electrode surface is the least rough. The *ƒ* for electrodeposited Pd-based electrodes increase in the following order: Pd-Pt < Pd < Pd-Rh < Pd-Ru. Moreover, Pd and Pd-Pt alloys are characterized by similar *ƒ* values, indicating the similarity of the real surface area to the geometric one. However, for Pd-Ru alloy, the *ƒ* value is ca. 37 times higher than for the Pd electrode. There is no clear correlation between *ƒ* value before and after corrosion test. The roughness factor significantly increases for Pd-Rh and Pd-Pt electrodes, whereas a slight decrease of the *ƒ* value is observed for Pd and Pd-Ru electrodes. The increase of the surface roughness factor after the corrosion test can be due to the creation of the large cracks (Figure 1). The presence of the large cracks leads to the exposure of the crystallites from the inner layers, which can contribute to the electrochemical processes. It can be seen that the electrochemical corrosion resistance parameters strongly correlate with the *ƒ* values, i.e., the electrodes with the largest real surface are the least resistant to corrosion. This effect is rather predictable because the size of the surface area of the sample with the highest ƒ value that interact with the electrolyte is much larger than that of the sample with the real surface area close to the geometric one. 

### 3.3. Scanning Kelvin Probe Measurements

**Figure 3:**
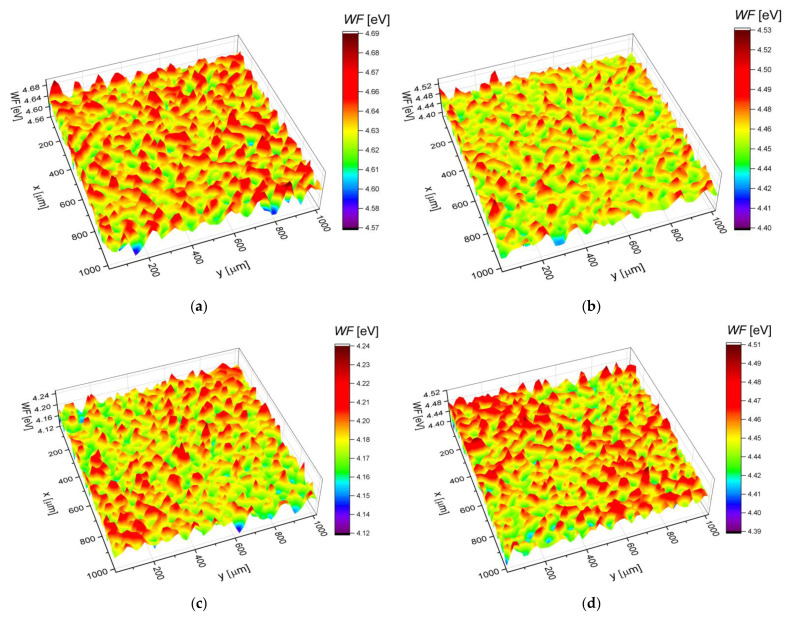
Work function maps determined for as-deposited electrodes: (**a**) Pd; (**b**) Pd-Rh; (**c**) Pd-Ru; (**d**) Pd-Pt.

**Figure 4:**
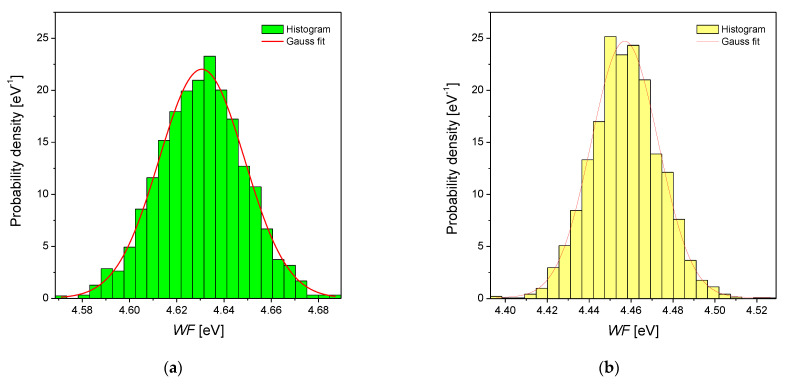

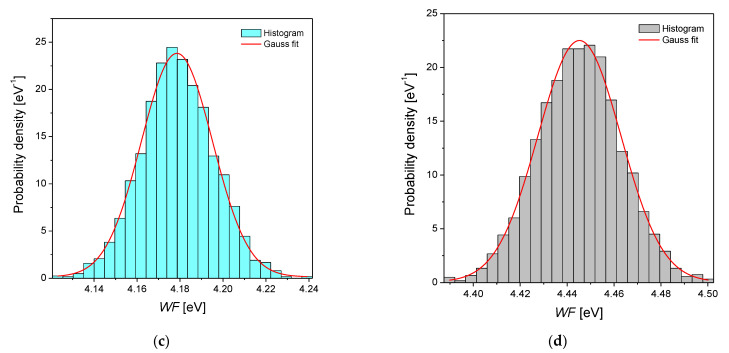
Distribution histogram of work function corresponding to Figure 3 for as-deposited electrodes: (**a**) Pd; (**b**) Pd-Rh; (**c**) Pd-Ru; (**d**) Pd-Pt. Continuous red lines represent fits obtained using the Gaussian function.

The measurements with the SKP technique have been done as a complementary method to the potentiodynamic measures. It allows the determination of *CPD*, which can be easily recalculated into the value of work function (*WF*). The *WF* definition concerns the difference between the Fermi level and the vacuum level [41]. The values of *WF* for the same material can be modified through: (i) carrier–doping, which results in the shifting of the Fermi level (*E_F_*) into higher values, and (ii) the modification of the surface dipole, which can increase the *WF* [41]. *WF* maps obtained for electrodeposited Pd, Pd-Rh, Pd-Ru and Pd-Pt electrodes are presented in Figure 3a–d and the corresponding histograms are shown in Figure 4a–d. Skewness and kurtosis are parameters that quantitatively describe peaks/valleys profile and provide information regarding the deviation of the work function distribution from a Gaussian distribution. It was found that skewness and kurtosis are in the range ±0.05 for all the investigated materials. Thus, the *WF* distributions follow a Gaussian distribution. Skewness and kurtosis close to zero indicate also that the *WF* values are symmetrically distributed around the average value and that there are no inordinately high peaks/deep valleys on the surface, irrespective of the alloying element. The obtained histograms were fitted by a Gaussian function:

(2) $$y\left(x\right)=\frac{1}{\sigma \sqrt{2\pi }}e^{-\frac{{\left(x-x_{av}\right)}^{2}}{2\sigma^{2}}},$$

where *x_av_* is the average value and *σ* is the standard deviation. 

Table 4 presents the average values of the work function (*WF_av_*) and standard deviation (*σ*) calculated based on the histograms, the literature values of *WF* for pure metals and alloys, and the values of the Fermi level energies calculated based on the approach proposed by Hara et al. [42] and values of *E_F_* of pure metals taken from the work of Papaconstantopoulos [43].

The calculated E*_F_* values for Pd and its alloys show that there is no significant difference in the position of the *E_F_* for the tested samples. In part, it results from the relatively low amount of the alloying metal (Rh, Ru, Pt). However, one can see that the position of E*_F_* is the lowest for Pd and the highest for Pd-Ru alloy, which is in agreement with the measured *WF_av_* and electrochemical data. According to Halas and Durakiewicz [44], for pure Pd, the E_F_ position, which equals ca. 7.69 eV, relates to the *WF* of ca. 5.12 eV. This value is higher than the one obtained from SKP measurements, which is ca. 4.63 eV. It is also worth noting that the surface oxides’ presence modifies the value of *WF* for every tested electrode. The above means that the *WF* theoretical value calculated for unoxidized metal/alloy in every case should be lower than the measured one since it is more difficult to extract an electron from an oxidized surface. Moreover, the XPS analysis of pure Pd and some of its selected binary alloys revealed that the Pd surface is oxidized in ca. 50%. The surfaces of alloy electrodes are oxidized in ca. 15–30% (depending on the type of alloy and its composition) [36]. However, in most cases, a few different effects have an impact on the *WF* value. Another parameter that influenced the value of *WF_av_* through the change of the position of *E_F_* is connected with the surface roughness of the tested electrodes. As mentioned above, the E*_F_* increase occurs when the surface dipole is modified, which follows in rough surfaces. For the Pd-Ru alloy with the highest *ƒ*, the value of *WF_av_* is the lowest. Onuferko et al. [45] and Losiewicz et al. [22] also relate the decrease of the *WF* with the increase of the surface roughness. *WF* values can also be different even for the electrodes of the same compositions but obtained by various methods. In Table 4, there is an example of a Pd_80_Rh_20_ electrode for which the value of *WF*_av_ is ca. 4.76 eV. This alloy electrode was obtained by arc–melting the Pd and Rh followed by annealing in argon in 650 °C. Assuming the *WF* linear correlation with the composition, it can be calculated that *WF* of the electrodeposited Pd-Rh electrode, containing 20 at.% Rh will be ca. 4.52 eV. This value is lower than for the arc–melted electrode. This example shows that *WF* value can also be modified by the preparation method of the electrode. It was found that the spread of the *WF* distribution around the average (represented by *σ*, Table 4) is comparable for investigated Pd-binary alloys. Moreover, standard deviation values indicate that ca. 95% of all measured *WF* values on the surface of Pd and Pd-binary alloys are in range *WF_av_* ± 0.03 eV; thus, *WF* varies slightly over the surface for all the investigated materials.

### 3.4. Electrochemical Impedance Spectroscopy

**Figure 5:**
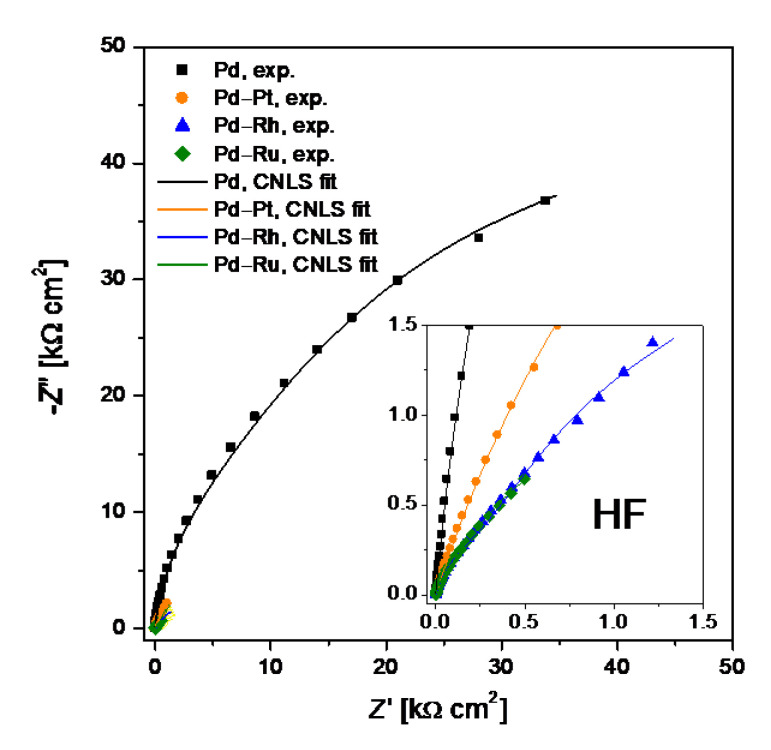
Complex–plane impedance plots obtained for electrodeposited Pd, Pd-Rh, Pd-Ru, and Pd-Pt electrodes in deaerated 0.5 M H_2_SO_4_ at the open circuit potential. Symbols and continuous lines represent experimental data and fits obtained using the equivalent electrical circuit shown in Figure 6 respectively.

Complex–plane impedance plots, −*Z*″ = *f*(*Z*′), recorded at the *OCP* for electrodeposited Pd and binary Pd-based electrodes in 0.5 M H_2_SO_4_ were presented in Figure 5. Two semicircles were visible for all tested electrodes. The diameter of the weakly developed semicircles at the high frequency (HF) range was significantly smaller than the diameter of the semicircles recorded in the low frequency (LF) range. 

The corresponding Bode plots in the form of impedance magnitude (log |*Z*|) and phase angle (*φ*) versus log frequency (log *f*) are shown in Figure 7a and b, respectively. The experimental values of |*Z*|*_f_*_→ 0_ and *φ* revealed that the tested electrodes were materials with capacitive behavior and high corrosion resistance. The values of log|*Z*|at the lowest frequency studied indicated deterioration of the barrier properties for binary Pd–based alloys compared to the Pd electrode (Figure 7a). Bode impedance plots indicated the occurrence of two time constants in Figure 7b. A wide plateau in the mid-frequency range for the Pd electrode confirmed its strongest barrier properties among all tested electrodes. The maximum values of *φ* are below −90°. The phase shift angle varies from 0 to −90° indicating capacitive behavior of all investigated electrodes (Figure 7b). Moreover, the plateau, which is observed in the relationship *φ* vs. the log *f* performed for Pd indicates that for this electrode the main process taking place at the electrode-electrolyte interface is charging the electrical double layer. Thus, for the Pd electrode, the rate of the charge transfer process through the electrode-electrolyte interface is low, which means good barrier properties of the oxides formed on its surface and, at the same time, high corrosion resistance of this electrode. The impedance modulus determined at *f* → 0 is comparable with the polarization resistance of the electrode. On this basis, it can be concluded that the resistance of oxides formed on the surface of the tested electrodes is the highest in the case of the Pd electrode, and thus the Pd electrode shows the best barrier properties. Among Pd alloys, the best barrier properties (the highest log |*Z*|) is shown by the Pd-Pt electrode.

**Figure 6:**
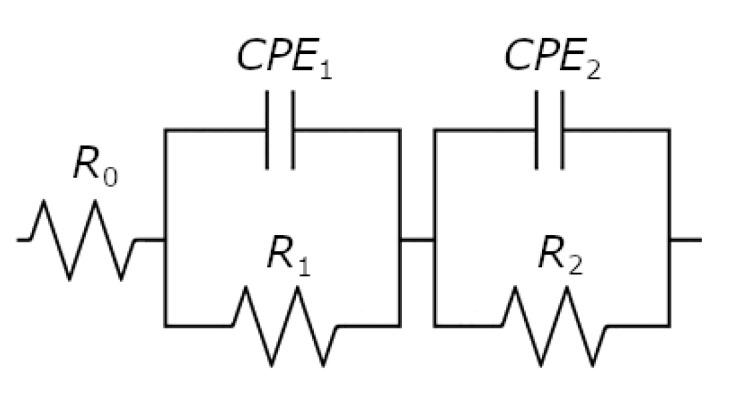
Equivalent electrical circuit model for the corrosion process used for CNLS-fitting.

**Figure 7:**
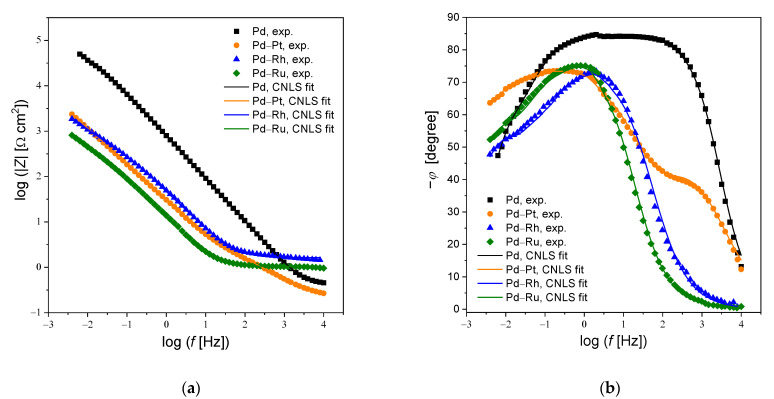
Bode plots versus log frequency (log *f*) for electrodeposited Pd, Pd-Rh, Pd-Ru and Pd-Pt electrodes in deaerated 0.5 M H_2_SO_4_ at the open circuit potential: (**a**) Magnitude of impedance (log |*Z*|); (**b**) Phase angle (*φ*). Symbols represent experimental data. Continuous lines are fits obtained using the equivalent electrical circuit shown in Figure 6.

The experimental EIS spectra were fitted using the equivalent electrical circuit shown in Figure 6. It is the so–called two–CPE model with five elements, i.e., *R*_0_, *R*_1_, *CPE*_1_, *R*_2_, and *CPE*_2_, where *R* and *CPE* denote resistors and constant phase elements, respectively [46]. *CPE* is an electrical circuit component that models a behavior of a double layer, which is an imperfect capacitor. It is due to the presence of physical, chemical, or geometrical inhomogeneities on the electrode surface [46]. The following equation gives the impedance of the *CPE* (${\hat{Z}}_{\mathrm{CPE}}$):

(3) $${\hat{Z}}_{\mathrm{CPE}}=\frac{1}{T{\left(j\omega \right)}^{\phi }}\;$$

where *T* is the capacitive parameter and *φ* determines the constant phase angle. Note that when *ϕ* = 1, then *T* = *C* and Equation (2) is the same as that for the impedance of a perfect capacitor. The components of the electrical circuit shown in Figure 6 assume a two-layer structure of the film (porous outer layer and tight inner layer) on the electrode surface. In this model, *R*_0_ represents the solution resistance, (*R*_1_*CPE*_1_) and (*R*_2_*CPE*_2_) can be associated with outer and inner layers, respectively. The obtained values of the *R*_0_, *T*_1_, *ϕ*_1_, *R*_1_, *T*_2_, *ϕ*_2_, *R*_2_ parameters are gathered in Table 5. 

It was stated that *ϕ*_1_ and *ϕ*_2_ parameters are <1, explaining the *CPE* instead of capacitor in the model. The comparison of *R*_1_ and *R*_2_ values indicates that electrical and geometrical properties of the inner layer decide about the corrosion resistance of all investigated electrodes. The highest *R*_2_ is observed for the Pd electrode. For Pd-binary alloys, *R*_2_ decreases in the following order: Pd-Pt > Pd-Rh > Pd-Ru. Generally, the values of *R*_2_ indicate that the corrosion resistance of Pd-binary alloys decreases compared to the Pd electrode. Parameter *T*_2_ shows that the decrease in Pd-binary alloys’ corrosion resistance is associated with an increase in the electrochemically active surface in the following order: Pd-Pt > Pd-Rh > Pd-Ru. The corrosion process of Pd alloys causes cracks at the surface of electrodes (Figure 1) and an increase in their electrochemically active surface, represented by *T*_2_ parameter. The access of the corrosive environment to the inner layer is then facilitated, which causes its degradation and a decrease in *R*_2_ resistance. The *R*_2_ values are comparable to the *R*_p_ parameter determined in the potentiodynamic measurements (see Table 2 and Table 5). A linear correlation was also found between capacitive parameter *T*_2_ and surface roughness factor ƒ determined after corrosion (see Table 3 and Table 5).

## 4. Conclusions

The electrochemical measurements of corrosion resistance parameters revealed that electrodeposited pure Pd is the most resistant to corrosion in deaerated 0.5 M H_2_SO_4_. It exhibits the highest value of the corrosion potential and polarization resistance and the lowest value of the corrosion current density. This result has been confirmed by the SKP method, which revealed that *WF* value is the highest for pure Pd compared to alloy electrodes. Based on EIS measurements, the capacitive behavior and high corrosion resistance of all materials were found. The AC impedance behavior of the tested electrodes may be well described using the electrical equivalent circuit containing two-*CPE* elements. It was confirmed that alloying Pd with relatively low amount of metals such as Rh, Ru, and Pt results in a deterioration of their resistance to corrosion in the following order: Pd > Pd-Pt > Pd-Rh > Pd-Ru. It was found that it is probably mainly due to the significant difference in surface roughness factor between pure Pd and its alloys. A huge impact of the surface roughness factor on the poor corrosion resistance of Pd-Ru electrode has been observed. 

## Figures and Tables

**Table 1:** Chemical compositions (EDX) of Pd-binary alloys before and after corrosion in at.%.

Alloy Type	As–Deposited	After Corrosion
Pd-Rh	96.9(4)% Pd 3.1(4)% Rh	97.0(1)% Pd 3.0(1)% Rh
Pd-Ru	96.3(3)% Pd 3.7(3)% Ru	96.4(3)% Pd 3.6(3)% Ru
Pd-Pt	96.7(7)% Pd 3.3(7)% Pt	94.7(3)% Pd 5.3(3)% Pt

**Table 2:** The corrosion resistance parameters for electrodeposited Pd and Pd-binary alloys in deaerated 0.5 M H_2_SO_4_ (see Figure 2).

Alloy Type	*j*_cor_ [µA cm^–2^]	*E*_cor_ [mV]	*b*_a_ [V dec^–1^]	*b*_c_ [V dec^–1^]	*R*_p_ × 10^4^ [Ω cm^2^]	*CR* at *E*_cor_ [mm yr^–1^]
Pd	0.26(1)	794.5(3)	0.096(1)	0.122(1)	9.0(3)	3.8(1) × 10^−3^
Pd-Rh	2.87(5)	733.6(3)	0.116(2)	0.066(1)	0.63(1)	4.08(7) × 10^−2^
Pd-Ru	10.1(1)	730.5(1)	0.159(2)	0.062(1)	0.19(1)	1.43(1) × 10^−1^
Pd-Pt	1.35(4)	748.5(4)	0.093(2)	0.068(2)	1.26(5)	1.92(6) × 10^−2^

**Table 3:** Surface roughness factor of the Pd and Pd-binary alloys before (*ƒ*_as–deposited_) and after corrosion (*ƒ*_after corrosion_).

Alloy Type	*ƒ*_as–deposited_	*ƒ*_after corrosion_
Pd	1.7(1)	1.3(1)
Pd-Rh	8.9(7)	15(1)
Pd-Ru	64(3)	58(3)
Pd-Pt	1.4(1)	10.4(8)

**Table 4:** Theoretical values of the Fermi level energy (*E*_F_) of the electrodeposited Pd and Pd-binary alloys and average work function (*WF*_av_) and standard deviation (*σ*) calculated using histograms shown in Figure 4a–d (*WF*_av_) as well as work function taken from the literature for alloys and pure metals (*WF*_lit_).

Alloy Type	*E*_F_ Calculated [eV] ^1^	*WF*_av_ [eV]	*σ* [eV]	*WF*_lit_ [eV]	Metal	*WF*_lit_ [eV] ^2^
Pd	7.06	4.63(1)	18.1(4) × 10^−3^		Pd	5.12
Pd-Rh	7.11	4.46(1)	16.0(5) × 10^−3^	4.76 ^3^	Rh	4.98
Pd-Ru	7.19	4.18(1)	16.5(5) × 10^−3^		Ru	4.71
Pd-Pt	7.11	4.45(1)	17.1(3) × 10^−3^		Pt	5.65

^1^ [42,43], ^2^ [44],^3^ for Pd_80_Rh_20_ [22].

**Table 5:** The value of parameters obtained using the equivalent electrical circuit shown in Figure 6.

Alloy Type	*R*_0_	*T*_1_	*ϕ*_1_	*R*_1_	*T*_2_	*ϕ*_2_	*R*_2_
	[Ω cm^2^]	[F cm^−2^ s*^ϕ^*^−1^]		[Ω cm^2^]	[F cm^−2^ s*^ϕ^*^−1^]		[Ω cm^2^]
Pd	0.44(1)	5.3(3) × 10^−^^4^	0.94(8)	8.4(7) × 10^3^	3.8(9) × 10^−^^4^	0.95(7)	82.9(9) × 10^3^
Pd-Rh	0.39(1)	91(9) × 10^−^^4^	0.88(6)	225(77)	90(9) × 10^−^^4^	0.81(3)	4.8(7) × 10^3^
Pd-Ru	0.43(1)	284(16) × 10^−^^4^	0.85(5)	167(12)	331(19) × 10^−^^4^	0.90(9)	2.0(7) × 10^3^
Pd-Pt	0.38(1)	76(9) × 10^−^^4^	0.69(2)	124(7)	77.1(4) × 10^−^^4^	0.80(1)	13.2(9) × 10^3^

## Data Availability

Not applicable.
